# AxoDetect: an automated nerve image segmentation and quantification workflow for computational nerve modeling

**DOI:** 10.1088/1741-2552/ad31c3

**Published:** 2024-03-28

**Authors:** David A Lloyd, Maria Alejandra Gonzalez-Gonzalez, Mario I Romero-Ortega

**Affiliations:** 1 Departments of Biomedical Engineering and Biomedical Sciences, University of Houston, Houston, TX, United States of America; 2 Jan and Dan Duncan Neurological Research Institute, Texas Children’s Hospital, Houston, TX, United States of America; 3 Department of Pediatric Neurology, Baylor College of Medicine, Houston, TX, United States of America; 4 Department of Biomedical Engineering, University of Arizona, Tucson, AZ, United States of America

**Keywords:** autonomic, simulation, neuromodulation, image segmentation, computer vision, FEM/FEA, NEURON

## Abstract

*Objective.* Bioelectronic treatments targeting near-organ innervation have unprecedented clinical applications. Particularly in the spleen, the inhibition of the cholinergic inflammatory response by near-organ nerve stimulation has potential to replace pharmacological treatments in chronic and autoimmune diseases. A caveat is that the optimization of therapeutic stimulation parameters relies on *in vivo* experimentation, which becomes challenging due to the small nerve diameters (2 $\mu{}$m), complex anatomy, and mixed axon type composition of the autonomic nerves. Effective development of *in silico* models requires tools which allow for fast and efficient quantification of axonal composition of specific nerves. Current approaches to generate such information rely on manual image segmentation and quantification. *Approach.* We developed a combined image-segmentation and model-generation software called AxoDetect: a target- and format-agnostic computer vision algorithm which can segment myelin, endo/epineurium, and both myelinated and unmyelinated fibers from a nerve image without training. *Main results.* AxoDetect is over 10 times faster on average when compared with current automatic methods while maintaining flexibility through the use of tunable pixel threshold filters to detect different types of tissue. When compared to a distribution-based and a manually segmented model of the splenic nerve terminal branch 1, the model generated with AxoDetect had comparable threshold prediction and was able to accurately detect an increase in activation threshold caused by the addition of surrounding fat tissue to the modeled nerve. *Significance.* AxoDetect contributes to the acceleration of neuromodulation treatment development through faster model design and iteration without requiring training. Furthermore, the computer vision approach and tunable nature of the filters in our method allow for its use in a variety of histological applications. Our approach will impact not only the study of nerves but also the design of implantable neural interfaces to enhance bioelectronic therapeutic options.

## 1. Introduction

Bioelectronic medicine uses electrical neuromodulation of autonomic peripheral nerves based on electrical pulse parameters designed for optimal activation or inhibition of target organ function for therapeutic purposes [1]. Treatment optimization considers the anatomical structure and location of such nerves, the type and material of the electrodes, and calculates depolarization thresholds using computational hybrid modeling; combining the finite element method (FEM) (for geometric reconstruction of the specific nerve tissue and bulk electromagnetic conduction simulations) with the NEURON simulation environment (for neurophysiological predictions of the activation and conduction of electrical impulses resulting from nerve stimulation) [2]. These hybrid modeling methods reduce the need for extensive empirical *in vivo* validation of nerve activation parameters and facilitate the prediction of effective therapeutic bioelectrical thresholds. Computational models have been developed to predict the activation thresholds in several clinically relevant targets including the vagus, sciatic, and splenic nerves [2–7]. In turn, the number, distribution and type of axons in these nerves have either been estimated, limited to a subjective minimum, or accurately represented based on transmission electron microscopy (TEM) histological data of the targeted nerve, with few studies focusing on small or unmyelinated fibers [3, 8, 9]. The incorporation of surrounding fat and vascular tissue to these computer models, which are resistive to electrical depolarization currents, has been recognized as an important aspect to include in the hybrid models for accurate nerve activation threshold prediction [9]. Given the importance of nerve tissue anatomy including surrounding tissue, we reasoned that computer models will benefit from incorporating an automated tissue segmentation process that can convert any nerve histology to an accurate FEM/NEURON model to simulate and predict activation threshold.

**Figure 1.**
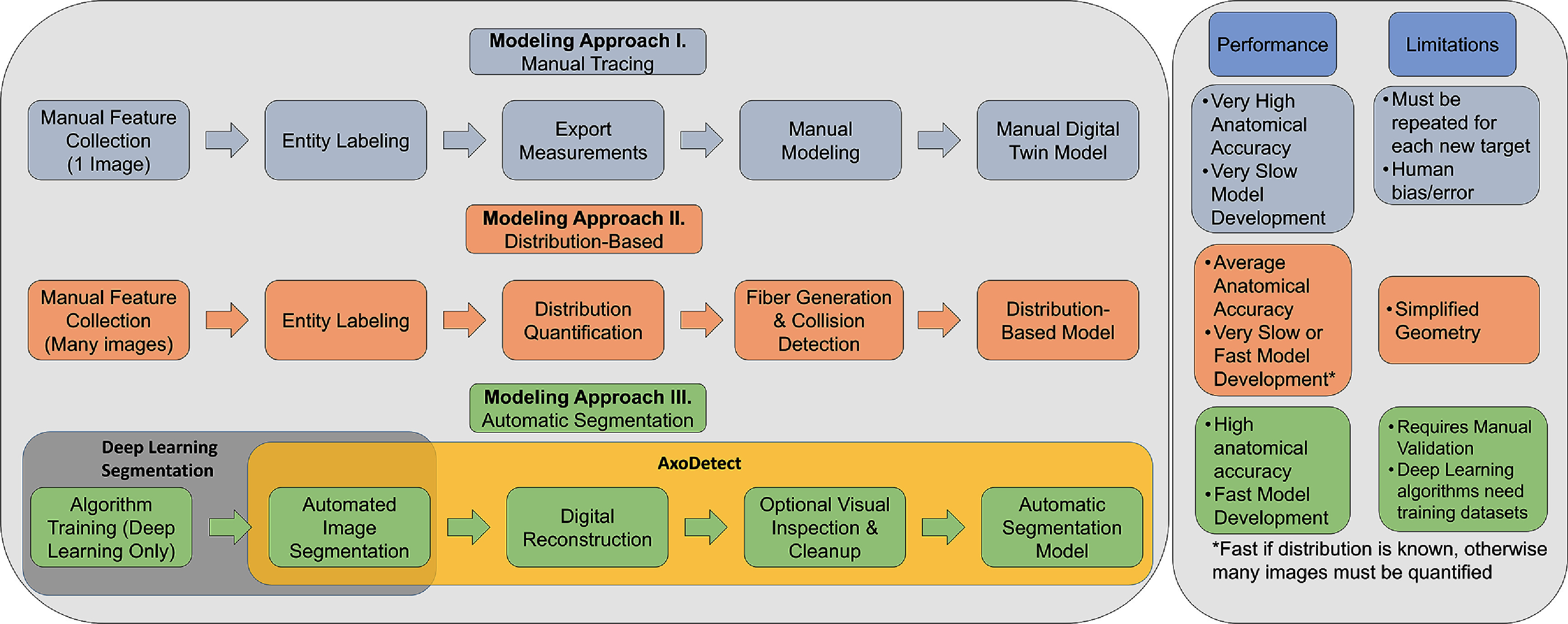
AxoDetect Workflow Comparison. In the left box, rows describe three types of image digitization approaches: Manual Tracing (blue), Distribution-Based (orange), and Automatic Segmentation (green), and their workflows. The right box lists their relative performance, anatomical accuracy, and limitations.

Tissue segmentation is currently done either by manual tracing (MT) or automatic segmentation (AS) (figure 1). MT models are constructed from manually labeled digitalized histology of individual tissue samples, which requires high-quality electron microscopy cross section images of the targeted nerve and accurate quantitative mapping of the axonal content of those nerves, to be performed by hand. Depending on the nature, size, and complexity of the nerve, an accurate and complete anatomical digitalization and quantification can take several hours to days. The AS approach, in contrast, uses software tools for axon segmentation and quantification, as well as tracing of other surrounding vascular and fat tissue from the nerve image samples. This process may be either supervised or unsupervised. Tissue segmentation might be obviated for nerves with substantially characterized morphologies, where instead a distribution-based (DB) modeling approach can be used. DB models are drawn from distributions of reported or pre-measured axon sizes, counts, and diameters for a particular nerve. DB models typically use simplified nerve composition and geometry to accelerate modeling, such as by generating perfectly circular axon cross sections rather than recreating fibers exactly [2].

Each approach brings its own set of limitations. MT models may introduce human bias and measurement error and are the least efficient to employ due to the segmentation and manual digitization required. The DB approach requires a defined distribution, and thus cannot be used to develop models for nerve targets that lack extensive characterization. The bulk of AS segmentation approaches rely on deep learning algorithms, which likewise requires transfer learning or re-training to apply them to new anatomical environments. Furthermore, these limitations are compounded by oversimplified anatomical detail such as (1) the endoneurium is often modeled as a bulk conductive material without discriminating individual boundaries and conduction values for myelin, fibroblasts, Schwann cells, endothelial cells, collagen, and axons, (2) unmyelinated fibers (UMFs) are often not included or assumed to be of a particular size and distribution.

Here, we introduce AxoDetect (AD), a fast, automated, and quantitative AS method designed to both segment nerve images and generate high-fidelity hybrid FEM models. AD can segment myelinated and UMFs, myelin, endoneurium area, and surrounding tissue (e.g. adipose, vascular tissue), and use these segmentation results to generate high-fidelity hybrid FEM. AD can segment images and generate models at a significantly higher speed than MT, DB, and other deep-learning-based AS approaches while maintaining equivalent accuracy to the gold standard approach. AD is a fast (i.e. less than 10 s) image-to-model workflow that uses computer vision (CV) through OpenCV in Python to segment and digitize nerve anatomy. It then digitally constructs a 3D model of the segmented nerve in Sim4Life (ZMT, v7.0) and applies bulk tissue conduction values to the different segmented tissue classes. AxoDetect can segment an image and generate a ready-to-simulate nerve model in seconds, in contrast to the hours or days that must be invested for other methods to perform segmentation alone. AxoDetect is novel in that it both performs the segmentation and generates a functionalized, ready-to-simulate model, compared to other approaches which only perform one of the two tasks [10–12].

Furthermore, since AxoDetect is a CV-based software, it requires no training and no extended dataset: only one single image is needed to generate a model. This is a substantial advantage for small or under-studied nerve targets which lack the bulk of image and morphometry data needed to develop a model with a deep learning AS approach or a DB approach. A comparative workflow diagram of the three image-to-model approaches is detailed in figure 1. While a few options for AS software are available (table 1), most are deep-learning-based and thus require large training datasets and transfer learning to achieve performance levels greater than 80% accuracy. In this study, we compare the segmentation quality and performance of AxoDetect against two advanced image segmentation protocols: 1) a ‘U-shaped convolutional network’ (hereafter referred to as UMF U-Net) for segmentation of unmyelinated fibers and 2) AxonDeepSeg for myelinated fibers [10, 11]. We also examine the threshold predictions for models of terminal branch 1 of the rat splenic nerve (Sp1) generated with MT, DB, and AS (AxoDetect) approaches.

**Table 1.** AxoDetect compared to two other AS Algorithms. Segmentation and performance are measured on the dataset from Havton *et al* [13]. All of these are automatic segmentation algorithms which do not require operator intervention. Optionally, visual inspection can be done on segmentation results for any of these methods.

Software	UMF U-Net	AxonDeepSeg	AxoDetect
Author	Plebani *et al* [10]	Zaimi *et al* [11]	Lloyd *et al*
Implementation	MATLAB	Python	Python
GUI	Yes	Yes, FSLeyes Plugin	No
Tissue segmented	Unmyelinated	Myelin, Myelinated	Myelin, Epineurium, Endoneurium, Unmyelinated, Myelinated
Methods	U-Net	Tensorflow CNN	Computer vision
Training required	Yes	Yes	No
Myelin segmentation time (s)	N/A	264.28 $+/-$252.11	22.39 $+/-$25.90
UMF segmentation time (s)	499.43 $+/-$448.06	N/A	61.99 $+/-$88.65
Myelin panoptic quality	N/A	0.01 $+/-$.009	0.11 $+/-$0.10
UMF panoptic quality	0.39 $+/-$0.15	N/A	0.11 $+/-$0.09

## 2. Materials and methods

### 2.1. Splenic nerve dissection

Four female Sprague-Dawley rats (300-350 g; Charles River, Wilmington, MA) were used in this study. SpN dissection was performed as described before [17]. In brief, vaporized isoflurane (2%) in a constant oxygen flux (2 l min^−1^) was used as anesthetic. The spleen was exposed by a 4 cm incision in the abdominal midline and located in the *upper* left portion of the abdominal cavity under the stomach. The four terminal neurovascular plexuses entering the hilum of the spleen (SpN) were dissected with sutures. An overdose of sodium pentobarbital (120 mg kg^−1^) was used for euthanasia, followed by intracardiac perfusion with physiological solution (PBS 1X, pH 7.4) and then by fixative solution (4% paraformaldehyde, 0.5% glutaraldehyde, in sodium arsenate (cacodylate) buffer, 0.1 M, pH 7.4). Afterwards the splenic terminal branches were harvested and processed for TEM imaging.

### 2.2. Ultrastructural analysis

The main splenic nerve and the four neurovascular plexuses of the spleen were processed for TEM. Samples were post-fixed in 3.0% glutaraldehyde in cacodylate buffer (0.1 M, pH 7.4) at 4 ^∘^C, and embedded in resin to obtain ultra-thin sections. Standard toluidine blue staining was performed on 700 nm semi-thin sections to evaluate structural organization. Ultra-thin sections of 60–70 nm were obtained using an ultramicrotome, mounted on copper grids, and coated with uranyl acetate and silver citrate for contrast. The sections were imaged using a transmission electron microscope (TEM, JEOL 1400 Plus, JEOL, USA).

### 2.3. Manual image segmentation

The TEM images of the SpNs were processed using FIJI v1.52p. From the gross anatomy, both, feature identification, and diameter measurements were delineated. Image contrast was improved using contrast limited adaptive histogram equalization with a block size of 127, 256 histogram bins, and a maximum slope of 3.00 in FIJI. The regions of interest (ROIs) were outlined with FIJI’s polygon selection tool and ROI manager function. A custom FIJI macro was written in Jython (v2.7.1) to extract the list of points which define each manually traced feature. The following features were identified, manually traced, and exported: unmyelinated fibers, myelinated fibers (exterior and interior bounds), fat cells, Schwann cells, blood vessels, epineurium, and endoneurium.

### 2.4. AxoDetect automatic image segmentation and model construction

**Figure 2.**
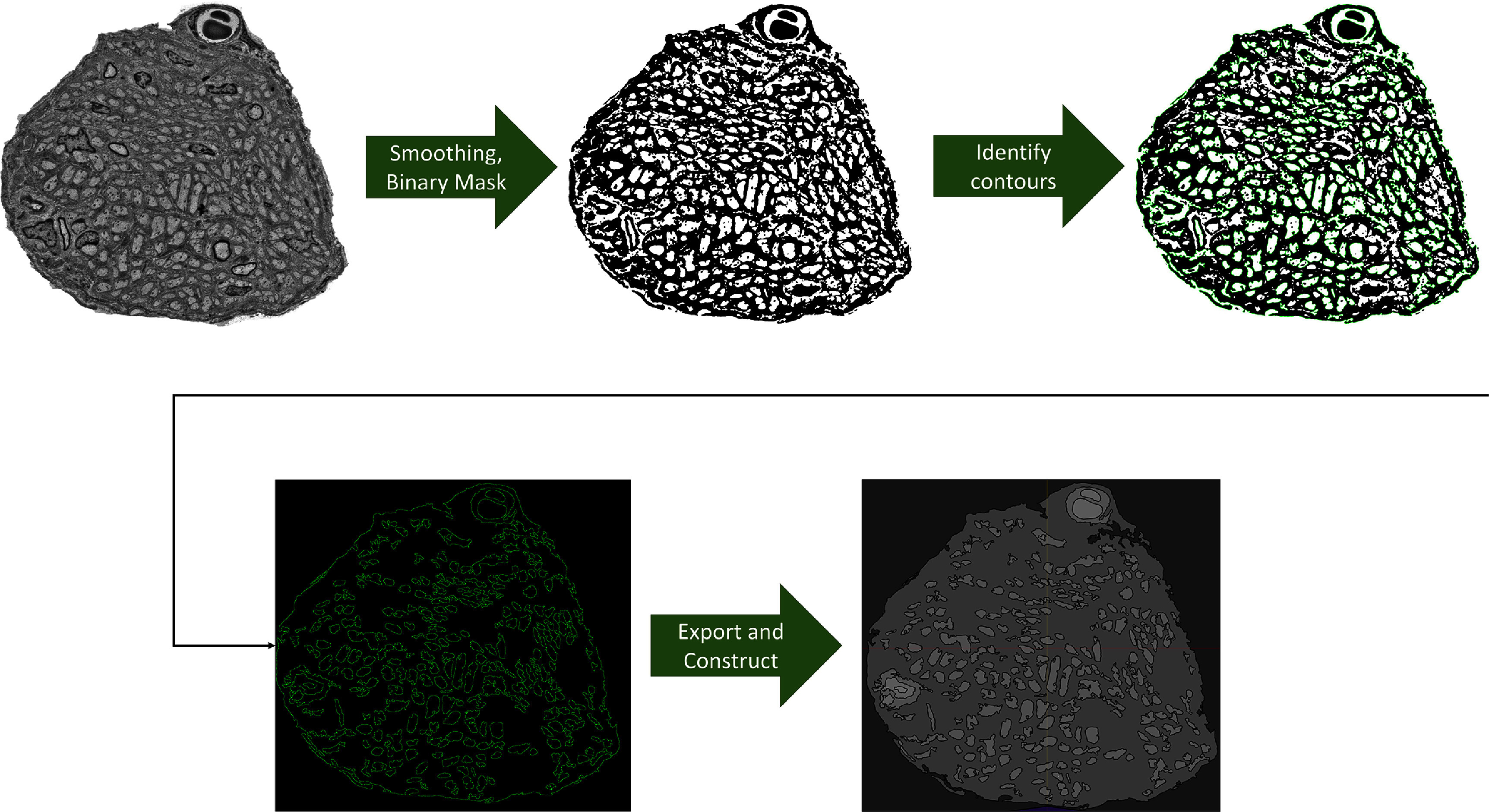
AxoDetect segmentation orkflow. Images were smoothed and converted to binary using different sets of filter banks to extract axons, epineurium, and other tissue.

Automated TEM image segmentation using AxoDetect was performed using OpenCV in Python. An 8x8 contrast limited adaptive histogram equalization was applied with a clip size of two bins to the input image. Equalized images were then processed with four different filters, which extracted different ranges of pixels. Each pixel intensity filter detected a different type of tissue (e.g. epineurium, myelin, and axons). Grayscale histograms were calculated for each image and a cumulative addition operation was used to identify intensity thresholds which best isolated structures of interest. The bottom 70% of pixels were filtered out to model the epineurium, the top 95% of pixels were removed to trace myelin sheets and Schwann cells, and both the top 30% and bottom 30% were removed to trace the axons within the TEM cross sections. OpenCV was used to detect the contours which were then filtered by circularity and contour length to determine shape eligibility. Circularity (*c*) was used as a filter to accept axons (i.e. circular in nature) from non-axon structures. Circularity *c* was defined as:

\begin{align*} c = 4\pi \left(\frac{A}{p^2}\right) \end{align*}

where *A* is the area of the shape and *p* is the perimeter length, with a perfect circle having a *c* value of 1. The characteristics that we used for axon tracing included: a contour length of 100 to 1000 points and a circularity of at least 0.1. For computational efficiency, all identified shapes were optimized with the Ramer–Douglas–Peucker algorithm with a tolerance (i.e. epsilon value) of 0.1%. The basic outline of the process is shown in figure 2. To generate the FEM model from the segmented image results, all detected shapes for each tissue class were imported and converted into swept 3D FEM models in Sim4Life (Zurich MedTech, v7.0) using the Sim4Life Python API. False positives (i.e. circular blood vessel detected as an axon) were visually inspected and removed inside the modeling software after segmentation. This process takes less than 2 min and consists of a brief visual inspection followed by selection and deletion of erroneous detections. AxoDetect automatically functionalizes model components with tissue conduction values (table 2) and fine-grained voxel-based discretization.

**Table 2.** Tissue conduction values: tissue conductivity values in $S/m$ that were used in simulations along with source. All conductivities were assumed to be isotropic.

Tissue	Conductivity ($S/m$)	Source
Fat	0.0673	[14]
Myelin	0.0010	[15]
Blood & blood vessel	0.6599	[14]
Saline	1.6000	[16]
Epineurium	0.1587	[8]
Endoneurium	0.2651	[14]

### 2.5. Segmentation quantification

Accuracy and speed of the AS were quantified; the panoptic quality (PQ) approach from [18] was used to compare segmentation accuracy with a multiplicative measure. Evaluation code was implemented in Python using OpenCV based on MATLAB code from [10]. PQ is a metric of the overall quality from 0 to 1 on how well the prediction and label masks match. PQ is a product of SQ (segmentation quality which quantifies detection of true positives) and recognition quality contains information on specificity of detection. Exact equation and method is outlined in [10, 18]. All of these metrics are calculated per instance (i.e. for each detected shape/contour). All quantification results and accuracies are reported without any intervention or segment exclusion.

### 2.6. Testing dataset

TEM images from the NIH SPARC repository by Havton *et al* [10, 13] were used to test all algorithms (a total of 21 sample images from 6 subjects). The dataset was used to train the UMF U-Net. Labels for myelin, fascicle area, myelinated fibers, and unmyelinated fibers were provided in XML text format from Neurolucida 360 software (MBF Bioscience, Williston, Vermont, USA) according to the FAIR file format for neuromorphological data [19]. Neurolucida software is licensed and proprietary, so a custom Python script was written to convert XML labels into binary mask images in .png format.

### 2.7. Parameter optimization

AxoDetect can be further improved by optimizing pixel intensity thresholds, circularity and size constraints, and preprocessing steps on a single image. This optimization was performed on image 131 F8 in the SPARC dataset using the COBYLA method with a basin-hopping algorithm. Initial conditions were given based on user determined values. The quantity ‘1 - PQ’ was minimized using this method to obtain the parameters used in the ‘opti umf’ filters.

### 2.8. Statistics

The Shapiro–Wilk test for normality and the Levene test for homogeneity of variances were used to verify that quality and performance data were non-parametric. The Friedman ranked sums test was used to compare performance among all segmentation filters and algorithms on the same set of images (matched samples) (figures 4 and 8) as well as the same models under different simulation conditions (figure 8), and pairwise comparisons were performed with the Wilcoxon signed ranks test using a Bonferroni correction. For comparison tests between modeling approaches, Kruskal–Wallis followed by Mann–Whitney–Wilcoxon with Bonferroni correction was used, as the study was not a matched design (figures 7 and 8). Significance level for all tests was set at $\alpha{}$ = 0.05. Statistical analysis was performed in Python using Pingouin [20], statistical annotations done with statannotations in Python [21].

### 2.9. Modeling assumptions

All models were made with a cross-section extrusion approach, and resultant nerves were assumed to be homogeneous throughout the 1 cm modeled length with zero tortuosity. Tissue conductivity was assumed to be isotropic, and conduction values are detailed in table 2. Stimulating electrodes were modeled as voltage sources and assumed to be perfect conductors. For DB models only, all neuromorphometric features were assumed to be circular. For the other model types, features were replicated according to the stated method. The system was considered to be quasi-static and Ohmic, and simulations were run with conductivity at 1 kHz. Neural simulations assumed a resting membrane potential of -80 mV. Tissue and axon temperature was assumed to be 37 ^∘^C. Unmyelinated axons larger than 1 $\mu{}$m were capped at 1 $\mu{}$m following limitations in the conduction model.

### 2.10. MT model

Sim4Life (Zurich MedTech, v7.0) FEM software was used for 3D modeling and both the electromagnetic and NEURON stimulations. Manually measured features were converted into the same format as AxoDetect’s segmentation results, and the model generation portion of the AxoDetect workflow was used to construct and set up a model from the MT segmentation. An arbitrary trajectory can be defined in the AxoDetect results-to-model functions by the user to match anatomical nerve fiber tortuosity. Outlines of measured ROIs were implemented as closed polylines, which were then swept along the given trajectory. This assumes that the size and location of all features is equal to that in the original cross section image throughout the length of the nerve. At the geometric center of each axon a spline is created following the overall nerve trajectory, which makes up the CAD portion of the hybrid model.

### 2.11. DB model

The means axon size and *g*-ratio and their standard deviations from [17] were used to generate a random number of myelinated and unmyelinated fibers for the four SpN terminal branches (Tb) in Sim4Life and NEURON (Zurich MedTech, v7.0). The total number of axons generated was equal to the average number of fibers per branch in the SpN Tb. After generating an endoneurium and epineurium, fibers were generated sequentially by drawing from the measured distribution, selecting a random location within the nerve, and checking 3D object collision values to ensure that the new fiber was colliding with the endoneurium, but not with the epineurium or any other nerve fibers. As before, splines for the Sim4Life NEURON simulation were generated at the geometric center of every. Two DB models were compared: DB and DBC. DB uses circular randomly generated axons but a MT of the epineurium and endoneurium. DBC uses a circular approximation of the epineurium and endoneurium instead of MT.

### 2.12. Bulk conduction simulation

**Figure 3.**
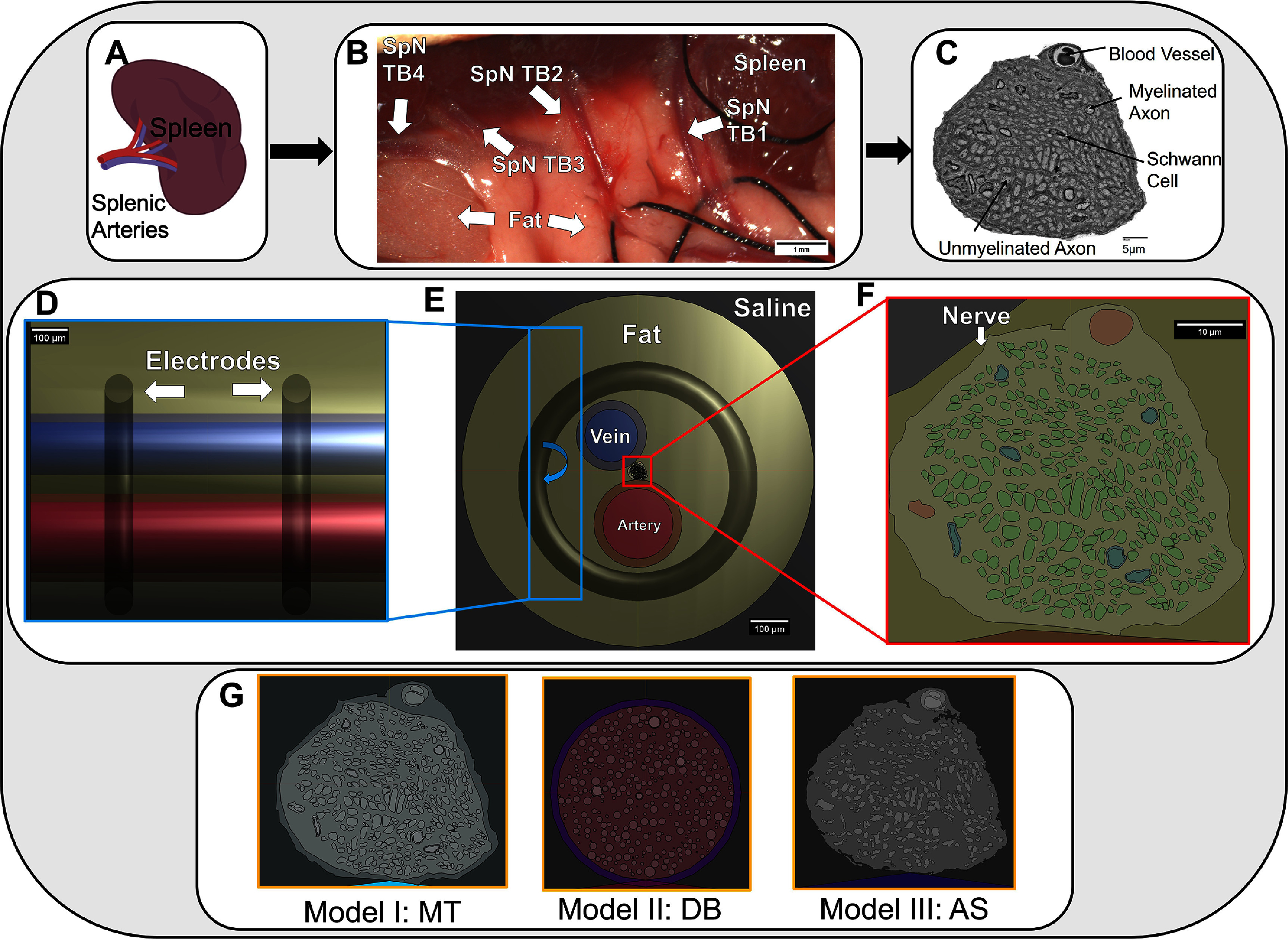
Anatomy of an FEM Nerve model. Panel A shows a cartoon of the spleen and innervating arteries, where the TBs enter the splenic body. Panel (B) is a surgical image of the 4 SpN TBs in their anatomical environment. Panel (C) is an example TEM image of SpN TB1 (SP1). Panels (D) and (F) branch out to show a rotated zoom and zoom on the electrodes and nerve respectively with a full cross section shown in Panel (E). Panel (G) shows an example cross section of nerve only for the 3 modeling approaches.

To build the electromagnetic (EM) simulation, Sim4Life’s Low Frequency (LF) Ohmic Quasi-Static solver was chosen to allow flexible, unstructured meshing for bulk conduction simulation. Anatomical structures and tissue types were identified and assigned properties as shown in table 2. A voxel-based mesh was implemented with a fine grid (maximum 0.001 mm) for neural elements and a coarse grid (maximum 1 mm) for other objects, with a total of 304 MCells generated, with priority voxeling. Voltage was applied by assigning each electrode a constant voltage Dirichlet boundary condition. Parallel bipolar microelectrodes with diameter 0.127 $\mu{}$m and inter-electrode spacing 0.5 mm were used as stimulating electrodes. Electrodes were placed next to SpN Tb1 at the edge of the fat in the model as shown in figure 3 Middle Panel. A stimulation with amplitude 100 $\mu{}$V (Vpp = 200 $\mu{}$V) was applied with the bipolar microelectrodes applied as a biphasic bipolar pulse of 250 $\mu{}$s or 1000 $\mu{}$s for each phase.

### 2.13. NEURON simulations

NEURON binaries for Sim4Life were used to perform recruitment studies. Unmyelinated fibers in the model were assigned the ‘Sundt’ model based on Sundt’s work in dorsal root ganglia electrical conduction [22]. For myelinated fibers, the Spatially Extended Nonlinear Node model was chosen due to the size of the fibers (1.8–3 $\mu{}$m in diameter), as the McIntyre-Richardson-Grill model is not suitable for axons with outer diameters smaller than 4 $\mu{}$m in diameter [23, 24]. Circular diameters were estimated for each fiber by dividing the contour perimeter by *π*. Bulk conduction results from the electrical simulations were interpolated onto NEURON trajectories, and a multiplicative titration factor *T* was applied iteratively until every fiber in the model had fired, and both spike time and titration factor at first firing were recorded for each fiber.

## 3. Results

### 3.1. AxoDetect improves accuracy and speed of myelinated fiber segmentation

On the images in the SPARC dataset with labeled myelin, nine different segmentation strategies were compared. 3 from AxonDeepSeg (myelin segmentation, myelinated fiber segmentation, and both overlaid) and 6 filters from AxoDetect. Average PQ for each segmentation algorithm across 15 images was compared with Friedman’s ranked sums followed by Wilcoxon pairwise comparison with Bonferroni multiple comparisons correction.

**Figure 4.**
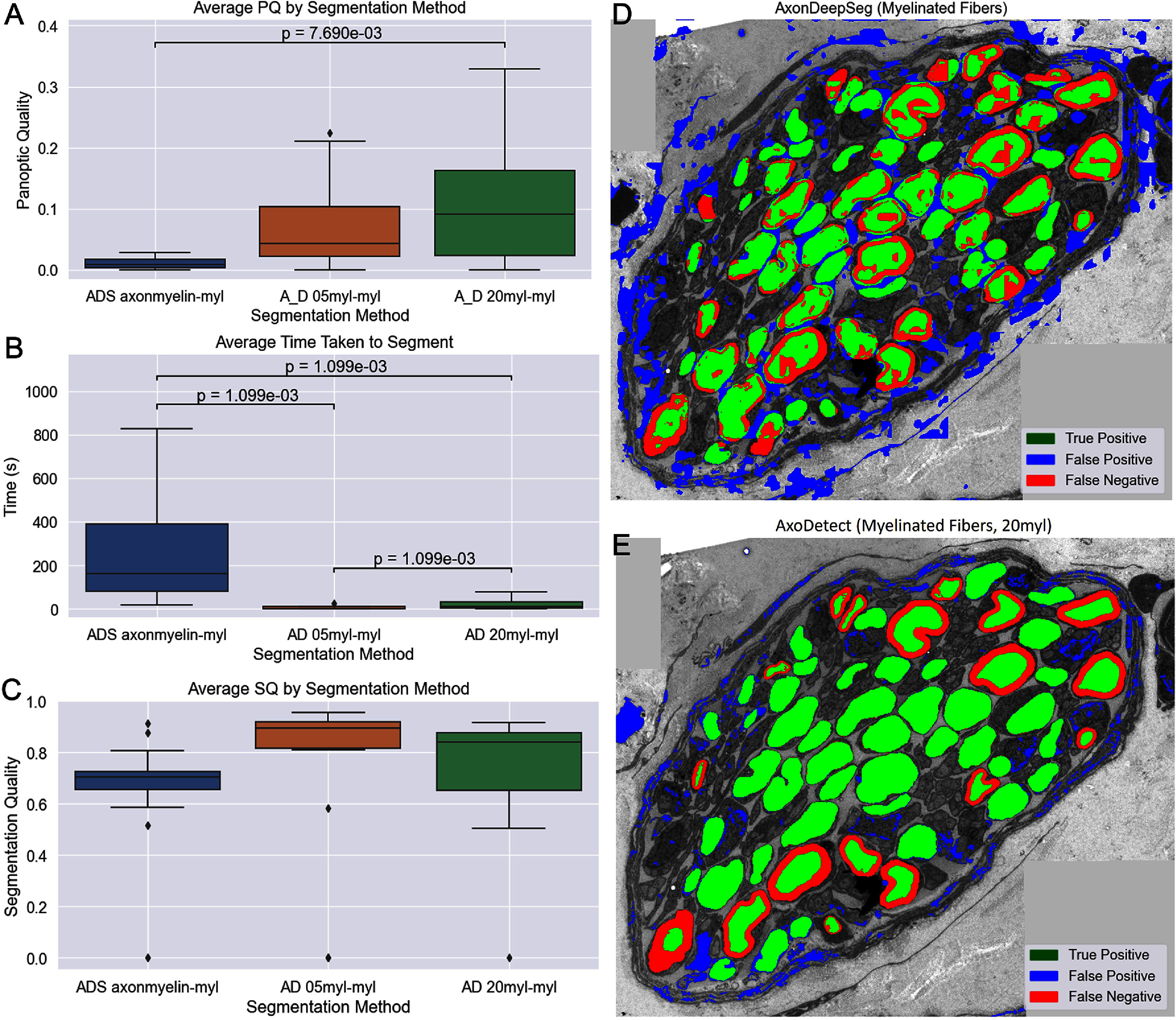
Myelinated Fiber Detection Performance. AxoDetect 20 myl filter shows significantly higher average PQ ($p = 7.69 \times $ $ 10^{-3}$) and significantly lower segmentation time ($p = 1.099 \times 10^{-3}$ for all pairs) compared to AxonDeepSeg from a pairwise Wilcoxon test with Bonferroni correction following Friedman’s test for 15 images. Panels (A) and (B) show the average PQ and segmentation time for only the two highest performing AxoDetect filters and the highest performing AxonDeepSeg prediction across the SPARC dataset. (C) shows the mean segmentation quality alone, where there is no significance between these groups. (D) and (E) show a representative image from the SPARC dataset with the AxonDeepSeg and AxoDetect predictions overlaid.

The top two AxoDetect and the top AxonDeepSeg results are illustrated in figure 4, and AxoDetect segmented images an average of 100-fold faster (*p* = 0.02) with a significantly higher panoptic quality (*p* = 0.04). Segmentation quality was not significantly different, however, indicating that the reduced PQ of the AxonDeepSeg process was primarily due to false positives and tiling artifacts which makes it difficult to create directly from AxonDeepSeg’s segmentation results. Despite this, AxonDeepSeg is highly tunable, so with some parameter optimization, performance could be improved substantially.

**Figure 5.**
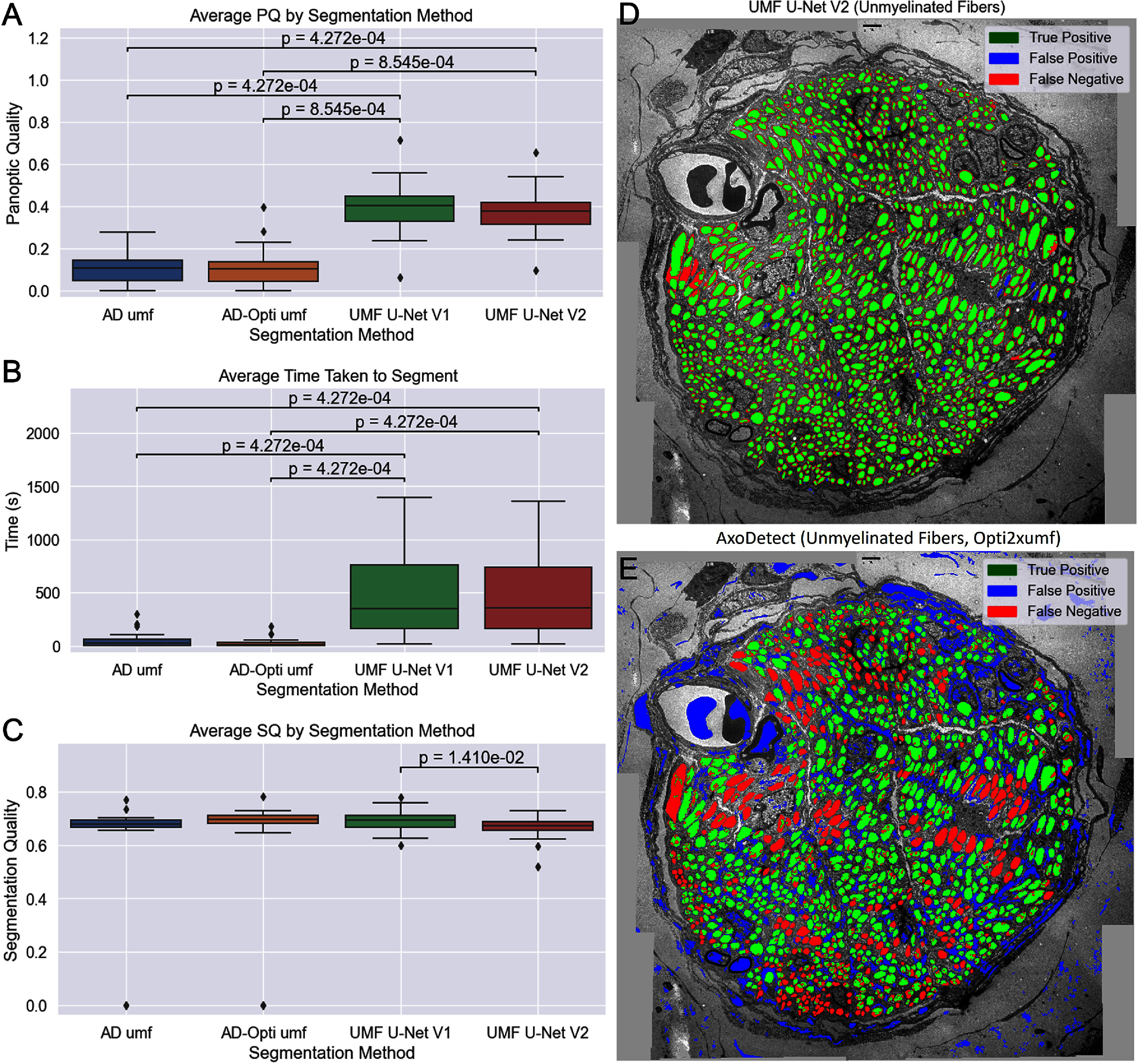
Unmyelinated fiber detection performance. AxoDetect umf and opti umf filters show significantly lower average PQ and significantly lower segmentation time compared to UMF U-Net V2 and UMF-U-Net V1 from a pairwise Wilcoxon test with Bonferroni correction following Friedman’s test for 17 images. Panels (A) and (B) show the average PQ and segmentation time for only the two highest performing AxoDetect filters and the UMF U-Net V2 and V1 predictions across the SPARC dataset. (C) Shows the mean segmentation quality alone, where there is only a significant difference between the two U-Nets. (D) and (E) show a representative image from the SPARC dataset with the UMF U-Net V2 and AxoDetect predictions overlaid. Marked significance is $p < \alpha < 0.05$.

The UMF U-Net was originally trained and developed on the same dataset employed for testing in this work, so it is expected to have excellent performance on these images. Both UMF U-Net V1 and V2 were compared with 8 filters from AxoDetect, including the optimized filters described in Methods. Average PQ, segmentation time, and SQ across 17 images in the dataset were quantified and compared with a matched nonparametric statistical approach (Friedman’s Ranked Sums followed by Wilcoxon pairwise comparison with Bonferroni correction). The top two AxoDetect and UMF U-Net results are illustrated in figure 5. AxoDetect showed significantly faster segmentation (*p* = 0.01) compared with the V2 U-Net, however the Panoptic Quality was significantly lower (*p* = 0.01) due to UMF U-Net being trained and optimized specifically for this dataset.

Segmentation quality was not significantly different between the two. This means the PQ difference was weighted by a lower RQ, reflecting the higher rate of false positives seen in AxoDetect when compared with the U-Net, which has an extremely low rate of false positives.

**Figure 6.**
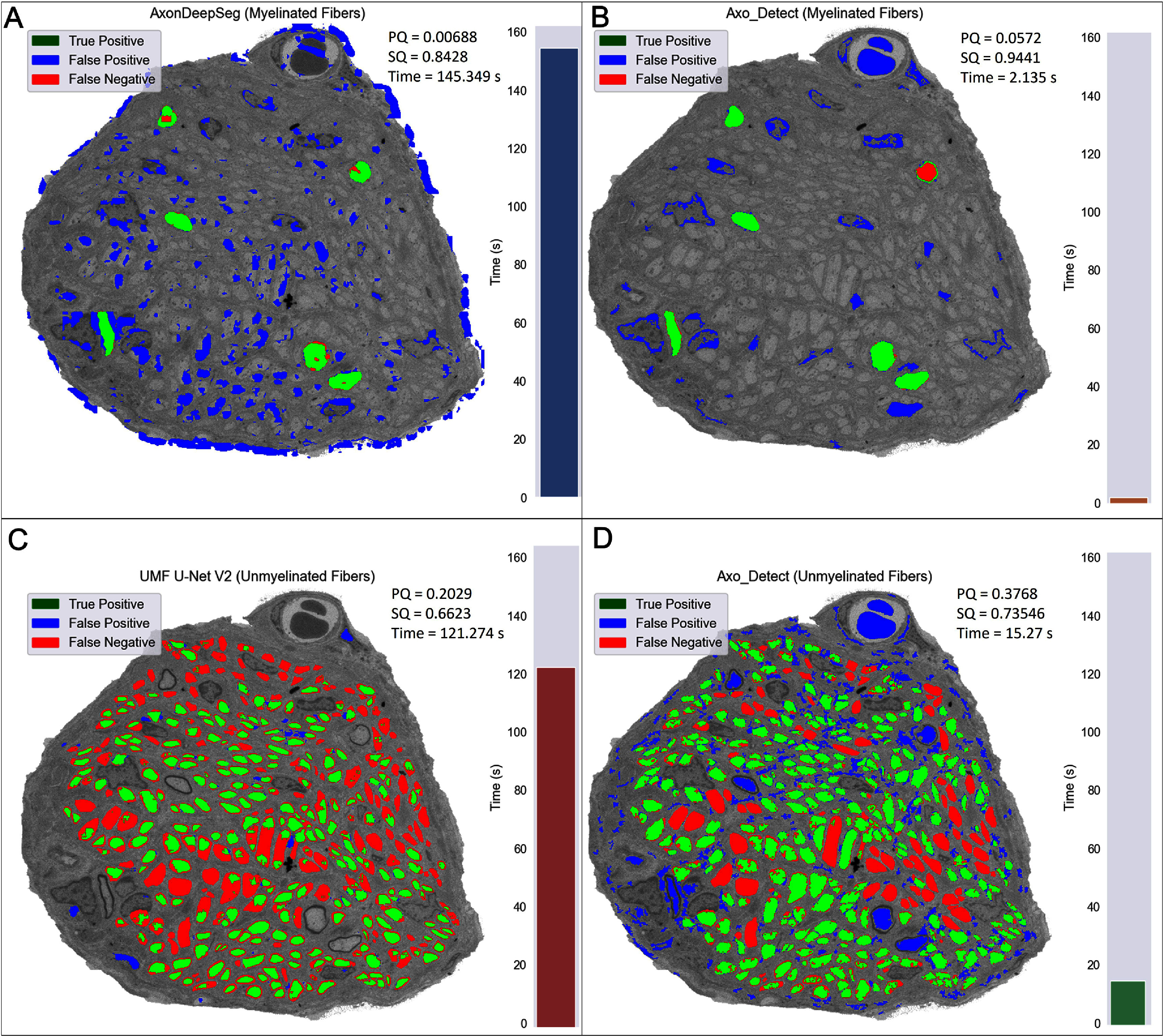
SpN Tb Performance of all algorithms. AxoDetect best performing filters for unmyelinated fibers (Panel (D) and myelinated fibers (Panel (B) compared UMF U-Net V2 without post-processing (Panel (C) for unmyelinated fibers and AxonDeepSeg for myelinated fibers (Panel (A). Panoptic quality and segmentation quality along with time to segment is noted on each of the images. Time required to segment the image is additionally indicated on a inlaid bar plot.

The primary driver for development of AxoDetect was for segmentation and modeling of nerve targets without large datasets available. With that in mind, performance was compared on a cross section of a terminal branch of the splenic nerve. Overlays with panoptic quality metrics are illustrated in figure 6 compared to manually measured results. Segmentation quality is comparable between the algorithms for both myelinated fiber detection and for unmyelinated fiber detection. AxoDetect was over 70 times faster for myelin segmentation compared to AxonDeepSeg, and over 8 times faster compared to UMF U-Net’s unmyelinated fiber detection for this particular sample.

### 3.2. AxoDetect model predictions are comparable to MT models

Titration simulations were run for each model with an initial voltage of 100 *µ*V, increasing stimulation voltage until 100% recruitment was achieved. The multiplicative scaling factor *T*, the titration factor, is the ratio between the required voltage to activate a fiber and the initial stimulus magnitude as $\phi_{T}(t)$ represents the electric field potential sensed by the neuron, $\phi()$ is the initial simulated electric field potential, *T* is the titration factor, and *a*(*t*) is the stimulation pulse waveform (balanced bipolar biphasic waveform).

\begin{align*} \phi_{T}\left(t\right) = \phi \cdot T \cdot a\left(t\right). \end{align*}

**Figure 7.**
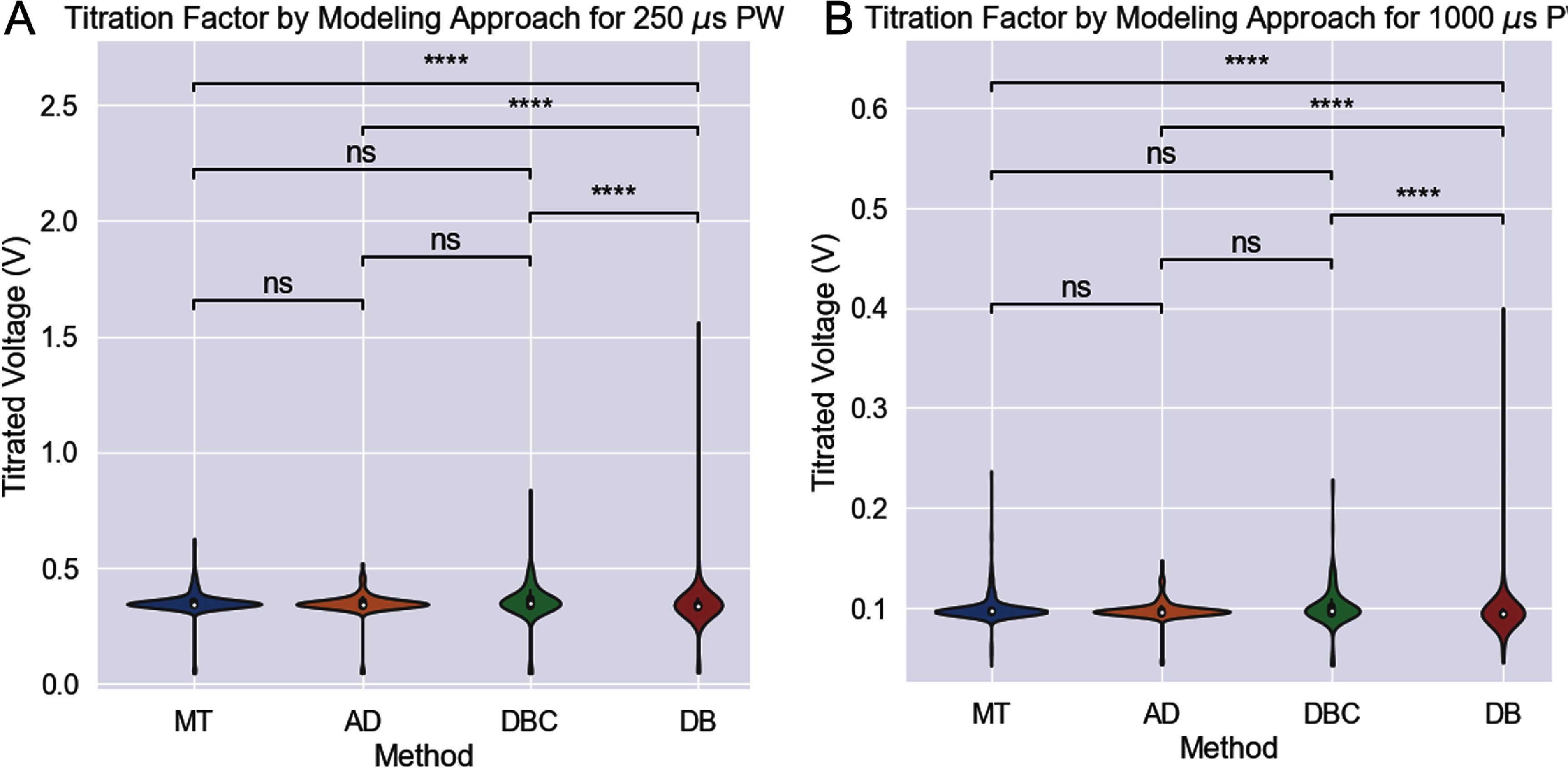
AxoDetect has statistically similar predictions to the MT model recruitment results for 3 different modeling approaches (with both a circular DB epineurium model, DBC, and a manually traced DB epineurium model, DB). Indicated *p*-values are from the Mann–Whitney U test following Kruskal–Wallis. Panel A shows the results for simulations with a 250 $\mu{}$s pulse width, while panel B shows results for simulations with a 1000 $\mu{}$s pulse width (bipolar, biphasic, balanced pulses).

Thus, titrated threshold voltage can be recovered by multiplying $\phi()$ by *T*. Recruitment curve kernel density estimates are calculated showing the ratio of fibers activated to total fibers over voltage. Figure 7 illustrates the predicted voltages needed to activate a model of SP1 from the 3 different modeling approaches. AxoDetect had statistically similar predicted voltage to the MT, which is the gold standard. The DB model on the other hand had statistically different predictions to all of the other approaches, indicating that this modeling approach was insufficient in this case to reflect the true anatomy of the nerve. The circular DB model had better performance, but likewise requires substantially more data and time to develop than that AD or MT model for a nerve target without an existing large dataset.

### 3.3. AD and MT models contrasted based on model components

As an example of a potential exploitation perspective for this work, eight simulations for the AD and MT model of Sp1 were compared. Threshold predictions were generated with and without visceral fat and surrounding veins and arteries. The resultant voltage predictions were compared and statistical analysis was performed according to section 2.8. A heatmap was developed to show significant and non-significant pairings in addition to the mean predicted voltages and predicted recruitment curves for each model. All pairs except two were significantly different, that being the MT and AD models with veins, arteries, and fat, and the MT and AD models with veins and arteries but no fat. However all other pairs were significantly different both within the MT or AD models and between modeling approaches.

## 4. Discussion

Accurate anatomical composition of autonomic nerves is needed for the preparation of computational models and simulations of neuron activation thresholds in bioelectronic therapies [8, 9, 25]. While several automated and deep-learning-based axon segmentation from electron microscopy histology approaches exist, most concentrate only on one type of fiber and focus on morphometric analysis rather than modeling [10, 11, 26]. Software which focuses more on segmenting unmyelinated axons has been recently developed, particularly based on axon size distribution analysis, rather than anatomically accurate number. Here we developed AxoDetect, an algorithm that can process fast and accurate automated axon cross section tracing and quantification, including recognition of blood vessels and fat tissue in the target nerve. This work is the first to present a non-deep-learning AS modeling approach with equivalent segmentation and simulation accuracy, high speed, and the ability to segment unmyelinated fibers, myelinated fibers, and other surrounding tissue with no training. This method is particularly useful for autonomic targets which are difficult to separate from surrounding tissue including those forming a neuro-vascular plexus, such as the intrabdominal, mesenteric, hepatic, enteric, pancreatic, and pelvic nerves. AxoDetect is uniquely poised to accelerate the development of more specific treatment methods by enabling fast, quantitatively accurate modeling, by enabling and facilitating the development of accurate in silico models for nerve targets which do not have substantial bodies of anatomical characterization. Particularly when results show better accuracy when compared to the gold standard MT method compared to the DB method (figure 7).

Another distinct advantage of the CV-based AxoDetect is that it can readily be applied to images of any modality and is not limited to just TEM. Since the pixelwise filters used in AD are easily customizable, color histology images such as heart muscle, solid organs and lung, are also possible to segment and quantify with the AD algorithm. Since AD does not need to be retrained it is reasonable to predict that control images (say, of normal nerves), can be compared directly in silico to individual histological sections of animals that underwent nerve injury, not only to quantify axon number, but to consider the entire nerve tissue in evaluating the degree of tissue regeneration, even beyond re-myelination [27].

Additionally, AxoDetect is designed to be modular. Other segmentation methods and simulation software can be integrated into the workflow to allow for increased flexibility for the users regardless of their system or target anatomy. So, while deep learning segmenters like AxonDeepSeg and UMF U-Net do not have any tools for modeling on their own, their outputs can be formatted to interface with the model construction portion of AxoDetect to extend their capabilities. On the other hand, the axon segmentation results from AD can be used as an input to other modular model-construction pipelines like ASCENT [12].

**Figure 8.**
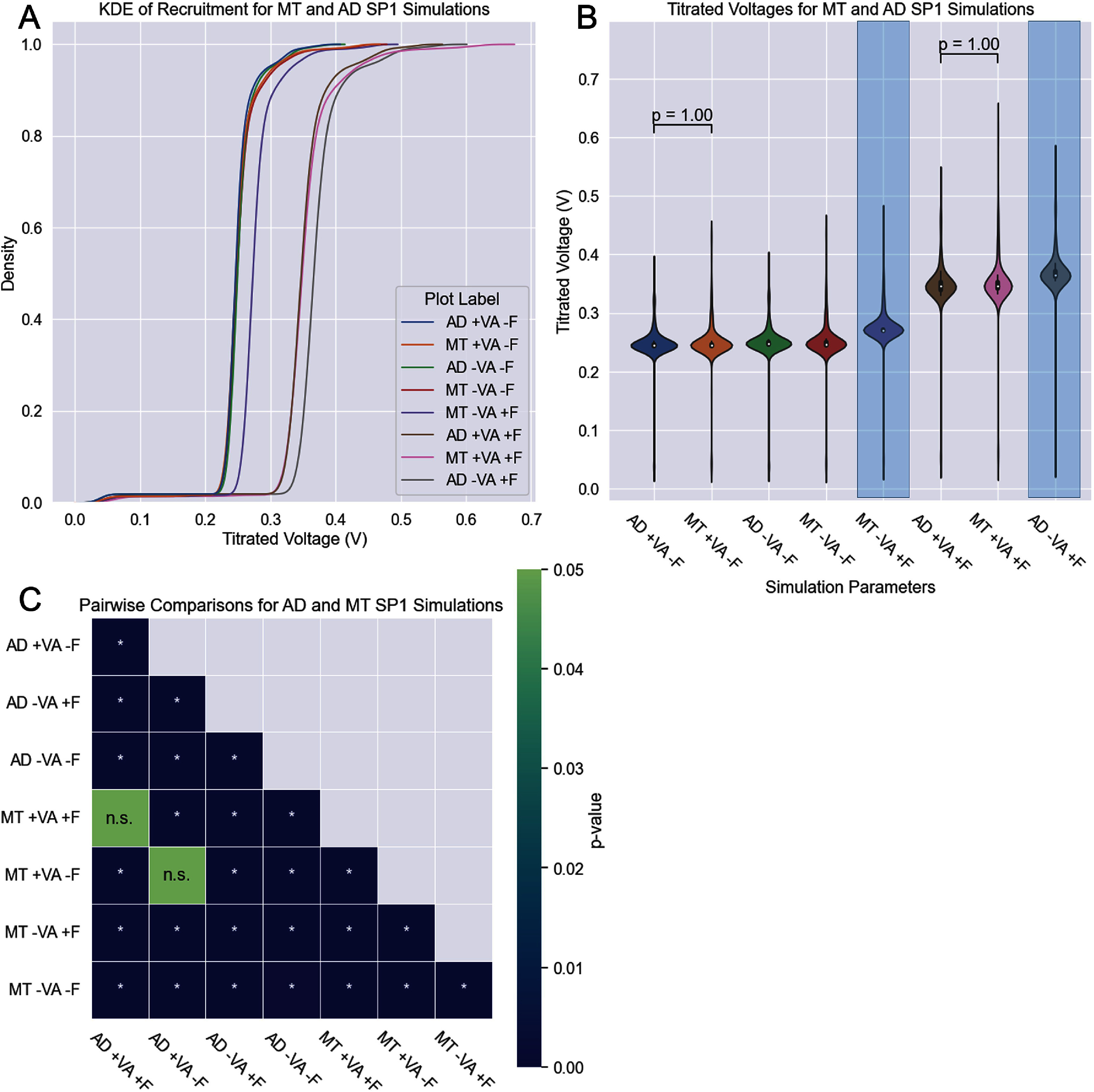
Changes in predicted threshold values in Sp1 models generated through AD and MT approaches. Panel A shows recruitment curves for Sp1 simulations with and without veins and arteries or visceral fat for both AD-generated and MT-generated models. Panel B shows the mean titrated voltage as a violin plot for each of these models and simulations. Panel C is a heatmap showing statistical comparisons for each simulation’s prediction. The *p*-values indicated are from the Mann-Whitney U test with Bonferroni correction. Due to the large number of significant pairs, only non-significant pairs are indicated on the graph: all pairs not indicated are significantly different. Full statistics are available in the supplementary materials.

AxoDetect is capable of both the image segmentation and model construction portions of the AS FEM modeling approach in one workflow. By combining this flexible design with open-source standards and file formats like those described by [19], these platforms and tools can have even farther reach in the development of next generation of neuromodulation predictive models. We also used AD to evaluate the importance of having an accurate anatomical composition of the nerve tissue, inclusive of blood vessels and fat, in the hybrid models for calculating nerve activation thresholds. When all anatomical components are considered, the model predictions are not significantly different as shown in figure 7. However, models in which tissue subcomponents are separated revealed a significant difference in almost all pairs between or within modeling approaches. The only pairs which are not significantly different are the MT and AD models which have veins and arteries but no fat (*p* = 1.00), and the MT and AD models that have both veins and arteries and fat (*p* = 1.00). In particular, when we only have visceral fat and no surrounding veins or arteries, there is over a 50% increase in the predicted activation threshold voltage between the AD and MT models (shown in figure 8, highlighted columns on panel B). Since the addition of fat as an insulation material is generally accepted as a factor that should increase the amount of depolarization energy needed, it seems that models that can evaluate the tissue subcomponents might offer a more accurate prediction of stimulation values. This is important, as a 50% underprediction in simulation energy thresholds has the potential to misinform power consumption and battery life requirements of implantable systems designed for some bioelectronic medicine applications.

In silico simulations for device design are especially relevant for targets such as the splenic nerve, which forms a neurovascular plexus that splits into terminal branches which travel in between blood vessels surrounded by adipose tissue and innervate the spleen. This makes these branches (numbering four in the rat) extremely difficult to dissect or directly interface *in vivo*. For in silico studies involving these or similar nerves, the model must reflect the location of the nerve in between the artery and vein and embedded in surrounding fat and connective tissue. This study not only emphasizes the importance of both intra- and extra-neural anatomy in hybrid nerve models, but also suggests that further studies are needed to characterize the effect that the employed model development approaches have on the model’s final predictive output. Neuromodulation of peripheral nerves is particularly relevant to those patients developing drug resistance for a given pharmacological therapy, and to reduce the systemic unspecific effects of pharmacological treatments. Particularly, chronic inflammation is associated with a cohort of clinical conditions, including rheumatoid arthritis and bowel disease [28–31]. AD has the potential to accelerate the generation of computational models of novel autonomic targets and to contribute to the accurate prediction of activation threshold energy needed for particular bioelectronic medical applications.

## Data Availability

The data cannot be made publicly available upon publication because they are not available in a format that is sufficiently accessible or reusable by other researchers. The data that support the findings of this study are available upon reasonable request from the authors.

## References

[1] Pavlov V A, Tracey K J (2022). Bioelectronic medicine: preclinical insights and clinical advances. Neuron.

[2] Romeni S, Valle G, Mazzoni A, Micera S (2020). Tutorial: a computational framework for the design and optimization of peripheral neural interfaces. Nat. Protocols.

[3] Pelot N, Musselman E, Marshall D, Davis C, Pena E, Hussein M, Huffman W, Shoffstall A, Grill W (2023). Advancing autonomic nerve stimulation through computational models. Brain Stimul..

[4] Schiefer M A, Triolo R J, Tyler D J (2008). A model of selective activation of the femoral nerve with a flat interface nerve electrode for a lower extremity neuroprosthesis. IEEE Trans. Neural Syst. Rehabil. Eng..

[5] Raspopovic S, Capogrosso M, Micera S (2011). A computational model for the stimulation of rat sciatic nerve using a transverse intrafascicular multichannel electrode. IEEE Trans. Neural Syst. Rehabil. Eng..

[6] Schiefer M A, Tyler D J, Triolo R J (2012). Probabilistic modeling of selective stimulation of the human sciatic nerve with a flat interface nerve electrode. J. Comput. Neurosci..

[7] Capogrosso M, Wenger N, Raspopovic S, Musienko P, Beauparlant J, Luciani L B, Courtine G, Micera S (2013). A computational model for epidural electrical stimulation of spinal sensorimotor circuits. J. Neurosci..

[8] Pelot N A, Behrend C E, Grill W M (2019). On the parameters used in finite element modeling of compound peripheral nerves. J. Neural Eng..

[9] Gupta I (2020). Quantification of clinically applicable stimulation parameters for precision near-organ neuromodulation of human splenic nerves. Commun. Biol..

[10] Plebani E, Biscola N P, Havton L A, Rajwa B, Sanjana Shemonti A S, Jaffey D, Powley T, Keast J R, Lu K-H, Murat Dundar M (2022). High-throughput segmentation of unmyelinated axons by deep learning. Sci. Rep..

[11] Zaimi A, Wabartha M, Herman V, Antonsanti P-L, Perone C S, Cohen-Adad J (2018). AxonDeepSeg: automatic axon and myelin segmentation from microscopy data using convolutional neural networks. Sci. Rep..

[12] Musselman E D, Cariello J E, Grill W M, Pelot N A, Schneidman-Duhovny D (2021). ASCENT (Automated Simulations to Characterize Electrical Nerve Thresholds): a pipeline for sample-specific computational modeling of electrical stimulation of peripheral nerves. PLOS Comput. Biol..

[13] Havton L A, Biscola N P, Plebani E, Rajwa B, Shemonti A, Jaffey D, Powley T L, Keast J R, Kun-Han L, Dundar M (2022). High-throughput segmentation of rat unmyelinated axons by deep learning. SPARC Consortium 2023.

[14] IT’IS Foundation (2018). Tissue properties database V4.0.

[15] Richardson A G, McIntyre C C, Grill W M (2000). Modelling the effects of electric fields on nerve fibres: influence of the myelin sheath. Med. Biol. Eng. Comput..

[16] Gawad S, Schild L, Renaud P (2001). Micromachined impedance spectroscopy flow cytometer for cell analysis and particle sizing. Lab Chip.

[17] Gonzalez-Gonzalez M A, Bendale G S, Wang K, Wallace G G, Romero-Ortega M (2021). Platinized graphene fiber electrodes uncover direct spleen-vagus communication. Commun. Biol..

[18] Kirillov A, He K, Girshick R, Rother C, Dollár P (2019). Panoptic Segmentation.

[19] Sullivan A E, Tappan S J, Angstman P J, Rodriguez A, Thomas G C, Hoppes D M, Abdul-Karim M A, Heal M L, Glaser J R (2021). A comprehensive, FAIR file format for neuroanatomical structure modeling. Neuroinformatics.

[20] Vallat R (2018). Pingouin: statistics in python. J. Open Source Softw..

[21] Charlier F, Weber M, Izak D, Harkin E, Magnus M, Lalli J, Fresnais L, Chan M, Markov N, Amsalem O, Proost S, Krasoulis A, Getzze, Repplinger S (2022). Trevismd/statannotations: V0.5. Zenodo.

[22] Sundt D, Gamper N, Jaffe D B (2015). Spike propagation through the dorsal root ganglia in an unmyelinated sensory neuron: a modeling study. J. Neurophysiol..

[23] McIntyre C C, Richardson A G, Grill W M (2002). Modeling the excitability of mammalian nerve fibers: influence of afterpotentials on the recovery cycle. J. Neurophysiol..

[24] Patrick Reilly J, Diamant A M (2011). Electrostimulation: Theory, Applications and Computational Model.

[25] Pelot N A, Goldhagen G B, Cariello J E, Musselman E D, Clissold K A, Ashley Ezzell J, Grill W M (2020). Quantified morphology of the cervical and subdiaphragmatic vagus nerves of human, pig and rat. Front. Neurosci..

[26] Christian Daeschler S C, Bourget M-H, Derakhshan D, Sharma V, Ivaylov Asenov S I, Gordon T, Cohen-Adad J, Howard Borschel G H (2022). Rapid, automated nerve histomorphometry through open-source artificial intelligence. Sci. Rep..

[27] Zoghoul Alsmadi N Z, Bendale G S, Kanneganti A, Shihabeddin T, Nguyen A H, Hor E, Dash S, Johnston B, Granja-Vazquez R, Romero-Ortega M I (2018). Glial-derived growth factor and pleiotrophin synergistically promote axonal regeneration in critical nerve injuries. Acta Biomater..

[28] Carey R M, Sakhuja S, Calhoun D A, Whelton P K, Muntner P (2019). Prevalence of apparent treatment-resistant hypertension in the united states: comparison of the 2008 and 2018 american heart association scientific statements on resistant hypertension. Hypertension.

[29] Nazarzadeh M, Pinho-Gomes A-C, Rahimi K (2019). Resistant hypertension in times of changing definitions and treatment recommendations. Heart.

[30] Watanabe R, Okano T, Gon T, Yoshida N, Fukumoto K, Yamada S, Hashimoto M (2022). Difficult-to-treat rheumatoid arthritis: current concept and unsolved problems. Front. Med..

[31] Fornaro R, Clemente Actis G C, Paolo Caviglia G P, Pitoni D, Giuseppe Ribaldone D G (2022). Inflammatory bowel disease: role of vagus nerve stimulation. J. Clin. Med..

